# Future Perspectives of Ectopic Pregnancy Treatment—Review of Possible Pharmacological Methods

**DOI:** 10.3390/ijerph192114230

**Published:** 2022-10-31

**Authors:** Milena Leziak, Klaudia Żak, Karolina Frankowska, Aleksandra Ziółkiewicz, Weronika Perczyńska, Monika Abramiuk, Rafał Tarkowski, Krzysztof Kułak

**Affiliations:** 1 I Chair and Department of Oncological Gynaecology and Gynaecology, Student Scientific Association, Medical University of Lublin, 20-081 Lublin, Poland; 2 I Chair and Department of Oncological Gynaecology and Gynaecology, Medical University of Lublin, 20-081 Lublin, Poland

**Keywords:** ectopic pregnancy, aromatase inhibitors, letrozole, methotrexate, KCl, absolute ethanol, gefitinib

## Abstract

Ectopic pregnancy, that is, a blastocyst occurring outside the endometrial cavity of the uterus, affects nearly 2% of pregnancies. The treatment of ectopic pregnancy is surgical or pharmacological. Since surgical management is associated with numerous serious side effects, conservative treatment is sought. The treatment of choice in the majority of cases is based on pharmacotherapy with methotrexate (MTX) in a single- or multi-dose regimen. Although the efficacy of methotrexate reaches between 70 and 90%, its use requires specific conditions regarding both the general condition of the patient and the characteristic features of the ectopic pregnancy. Moreover, MTX can cause severe adverse effects, including stomatitis, hepatotoxicity and myelosuppression. Therefore, clinicians and researchers are still looking for a less toxic, more effective treatment, which could prevent surgeries as a second-choice treatment. Some studies indicate that other substances might constitute a good alternative to methotrexate in the management of ectopic pregnancies. These substances include aromatase inhibitors, especially letrozole. Another promising substance in EP treatment is gefitinib, an inhibitor of EGFR tyrosine domain which, combined with MTX, seems to constitute a more effective alternative in the management of tubal ectopic pregnancies. Other substances for local administration include KCl and absolute ethanol. KCl injections used in combination with MTX may be used when foetal heart function is detected in cervical ectopic pregnancies, as well as in heterotopic pregnancy treatment. Absolute ethanol injections proved successful and safe in caesarean scar pregnancies management. Thus far, little is known about the use of those substances in the treatment of ectopic pregnancies, but already conducted studies seem to be promising.

## 1. Introduction

Ectopic pregnancy (EP), also known as extrauterine pregnancy or eccysis, refers to an implantation of a developing blastocyst outside the endometrial cavity of the uterus [1]. The frequency of ectopic pregnancies is estimated at nearly 20 per 1000 confirmed pregnancies [2]. The risk factors are the following: multiparity, previous EP episode, intra uterine device (IUD) used before conception, abdominal surgeries and the use of artificial reproduction techniques [3,4]. A total of 90% of EPs are located in the fallopian tube; the remaining 10% can occur in the abdominal cavity, cervix, ovary, interstitial portion of the fallopian tube, broad ligament, the uterine cornea, or within a caesarean section scar [4].

There is no single effective treatment for EP; the appropriate management depends on its location and the patient’s condition. Therapeutic methods used in this pathology include both pharmacotherapy and a wide range of surgical techniques [5]. Since a certain number of EPs undergo spontaneous reabsorption, one way to proceed is expectant management: observation of the patient with monitoring of the β-human chorionic gonadotropin (β-hCG) levels. Indications for the use of pharmacological management including a single- or multi-dose regimen of methotrexate (MTX) administration are the atypical localization of EP and contraindications for the surgery treatment [5]. Although the multi-dose protocol is associated with greater efficacy, it also causes more side effects [6]. If the patient is not eligible for MTX therapy, pharmacology proves ineffective, or if life-threatening complications such as rupture of the fallopian tube develop, surgical treatment is necessary [7]. It is also the method of choice in the case of heterotopic pregnancy, in which one of the embryos is implanted ectopically and the other in the uterine cavity. Since EP is both a leading cause of maternal mortality in the first trimester [8] and there are many side effects of MTX and surgical treatment, there is a great need for the development of a new therapy. The aim of our study was to present other, less-known medical methods for the treatment of EP that might replace methotrexate therapy.

## 2. Materials and Methods

The studies cited in the presented review were selected from PUBMED, Google Scholar and Science Direct databases. The research was conducted from November 2021 to October 2022. The key words used for the search included: “ectopic pregnancy treatment”, “letrozole and ectopic pregnancy”, “gefitinib and ectopic pregnancy”, “KCl and ectopic pregnancy” and “absolute ethanol and ectopic pregnancy” (Supplementary Materials). We included studies from 2000 to 2022. Articles not written in English, conference abstracts only and duplicated papers were excluded. 

## 3. Treatment with Methotrexate

The pharmacological treatment of ectopic pregnancy is mainly based on the administration of methotrexate [5]. Its action is based on the inhibition of DNA synthesis at various stages of the cell cycle and, in consequence, the death of rapidly dividing cells including trophoblast cells. This mechanism led to the use of methotrexate in the treatment of ectopic pregnancy [9,10]. Although the efficacy of methotrexate in the treatment of ectopic pregnancy reaches approximately between 70 and 90% depending on the type of treatment regimen used, the use of methotrexate requires specific conditions, both regarding the general condition of the patient and the characteristic features of the ectopic pregnancy. For the patient to be qualified for the methotrexate treatment, neither symptoms indicating hemodynamic failure nor a number of other symptoms concomitant with fallopian tube rupture can be present [11,12,13]. Furthermore, the coexistence of clinically significant liver or renal diseases, bone marrow dyscrasias, immunodeficiency, peptic ulcer disease, breastfeeding and the coexisting intrauterine pregnancy precludes the administration of methotrexate to patients due to the elevated risk of developing adverse reactions [12,13].To increase the effectiveness and safety of the methotrexate treatment outcome, the serum β-hCG level should be lower than 1500 IU/L, the foetal heart rate should be undetectable, and the size of the gestational follicle should not exceed 35 mm in size [13]. Other studies also indicate success in MTX treatment with β-hCG values below 4000 IU/L [14,15]. Moreover, β-hCG level should be regularly assessed, as its increase above the normal limit as well as an initial β-hCG level above the normal range have shown to correlate with an increased risk of a dangerous complication of methotrexate treatment, i.e., fallopian tube rupture. This complication occurs in 7 to 14% of women with ectopic pregnancy treated with methotrexate. Thus, the inability to monitor the patient’s condition constitutes another contraindication to methotrexate therapy, and when an observation of the patient is not possible, such treatment should not be implemented [13,16,17]. Although methotrexate is a relatively safe substance, its administration may be associated with numerous adverse effects such as nausea, upset stomach, diarrhoea, stomatitis, fever, headache, fatigue, hepatotoxicity and myelosuppression [10,18]. Side effects generally affect approximately 37% of treated women. Additionally, there are reports indicating that a multiple dose of methotrexate therapy is associated with a higher incidence of side effects than a single-dose administration [19].

Due to limitations in the use of methotrexate in a large group of patients and its serious adverse effects, it is necessary to seek other competitive methods for the pharmacotherapy of ectopic pregnancy.

## 4. Aromatase Inhibitors—Letrozole

The aromatase inhibitors’ (AIs) action is based on the inhibition of androstenedione and testosterone to estrone and oestradiol conversion; further, the suppression of oestrogen production is observed [20].

The idea of using AIs in the treatment of EP is inseparably associated with the undeniable role of oestrogens in the process of implantation and embryonic development [21]. Moreover, some evidence indicates that the inhibition of aromatase enzymes may result in insufficient progesterone influence on maintaining early pregnancy. Hence, the authors stated that the reduction in oestrogen production might lead to a decrease in the number of progesterone receptors [22].

The impact of AIs on pregnancy was observed on animal models, in which early development of the embryo was disturbed [23] and the maintenance of pregnancy was not possible [24]. Several studies, including a meta-analysis conducted on 555 women, showed the high efficacy of AIs administered in a combination with misoprostol in abortion treatment [25].

Kochhar et al. described the case of a 28-year-old woman after two caesarean sections. At 11 weeks of pregnancy, the woman reported irregular vaginal bleeding, dull pain that was present for 3 weeks, and dizziness. β-hCG was found to be 1525.51 mIU/mL. A diagnosis of chronic (the form of tubal pregnancy in which the attachment site of the trophoblastic tissue is destroyed, leading to bleeding and the formation of a haematocele [26]) EP was confirmed by ultrasound examination. The patient was treated with letrozole, a dose of 7.5 mg daily for 4 days, and then 5 mg daily for 3 days, taking 2.5 mg tablets. Treatment effectiveness was determined by β-hCG measurements and by transvaginal ultrasound (TVS). The β-hCG level was measured before the start of the treatment, and when measured after 4 and 7 days as the treatment progressed, the levels declined. TVS proved no free fluid, no rupture mass, and the size decreased [27].

Mitwally et al. conducted a non-randomized study evaluating the effect of letrozole on EP compared to MTX. The study was performed on 42 women with a tubal ectopic pregnancy (tEP). The women were divided into three groups of 14 people each. The control group was treated with salpingectomy; the other two groups were treated with MTX and letrozole separately. The MTX group received one intramuscular injection of 50 mg/m^2^ of MTX, while the letrozole group received 5 mg of letrozole in two tablets of 2.5 mg each daily for 10 days. β-hCG level was measured four times: on the first day of the treatment, and on the fourth, seventh, and fourteenth day after the treatment. Additionally, the level of haemoglobin and platelets, kidney function, liver enzymes, and the antimüllerian hormone (AMH) were measured to determine the possible negative influence of MTX or letrozole. EP was completely terminated in 86% of patients in both study groups. There was no significant difference in β-hCG levels in the two groups on the day of the initiation of treatment, while β-hCG levels began to decline more rapidly in women treated with letrozole than with MTX. In the MTX group, the authors observed a decrease in haemoglobin and platelets levels, and an increase in the levels of liver enzymes. Letrozole, compared to MTX, did not affect the parameters determined in the study. Letrozole also did not affect AMH levels 3 months after treatment, while MTX reduced it, but the result was not statistically significant [22,28].

In 2022, El-Sayed at all. published the results of a non-randomized study involving 50 women with an average age of 28 years who were diagnosed with tubal ectopic pregnancy. The patients were divided into two groups, one treated for 10 days with letrozole (2.5 mg) twice daily and the other with MTX 1 mg/kg. The results of the studies show that both treatments were equally effective with success rates of 86%, with β-hCG levels concentrations decreasing more rapidly in women treated with letrozole [29].

## 5. Gefitinib

Gefitinib is an inhibitor of the epidermal growth factor receptor (EGFR) tyrosine kinase domain and, as a result, it blocks receptor-controlled growth pathways [30]. Currently, this medicine is widely used to treat non-small cell lung cancer [31].

The first reports of the possible use of gefitinib in EP treatment appeared in 2013 when Nilsson et al. conducted a preclinical study using this substance [32]. The attempt to use gefitinib to treat EP was motivated by previous reports concerning the high expression of the EGFR in placental tissue compared to other non-malignant tissues, and by the dependence of placental cell growth on the EGFR-mediated pathways [33,34]. The results of an above-mentioned study showed that the growth of placental cells in vitro was most effectively inhibited by a combination treatment of gefitinib and MTX when compared with the separate administration of these substances. Moreover, similar observations suggesting an effective combination of these two substances have been demonstrated on animal models. Thus, the combined usage of gefitinib and MTX in immunodeficient mice with implanted human placental cell xenografts resulted in a greater reduction in xenografts volume when compared with the separate use of these agents. Moreover, in immunocompetent mice, the administration of combined therapy resulted in approximately twice as frequent foetal resorption as methotrexate and gefitinib alone [32].

The promising results of this preclinical study led to several studies assessing the effectiveness of the combined therapy of gefitinib and MTX in women with EP. All of these studies confirmed the effectiveness of the use of such therapy in the treatment of EP. In the first clinical trial, Skubisz et al. analysed the combination therapy in twelve women with EP with a β-hCG pre-treatment range between 1000 and 3000 IU/L. They observed that the complementation of MTX treatment with gefitinib resulted in a greater reduction in β-hCG levels. Moreover, such combined therapy proved to progress the resolution of EP by 11 days on average when compared with women from the control treated with MTX alone. Importantly, combination therapy was effective in ten out of twelve patients receiving this treatment, which means that it was successful in 85%. Additionally, no serious adverse reactions were observed in patients treated with gefitinib [35].

Another clinical trial conducted in a larger group of 28 patients presented the same conclusions concerning the efficacy of combined therapy. They noticed a comparable percentage (86%) of effectiveness of the combination therapy [36]. Similar to the previously mentioned study [35], no serious adverse effects related to the gefitinib administration were observed. In contrast to the previous report, the pre-treatment serum β-hCG values ranged from 1000 to 10,000 IU/L. High efficacy in the high β-hCG levels group suggests that this method can be used in ectopic pregnancies characterized with higher β-hCG levels [36].

EPs located outside the fallopian tube represent a significant therapeutic challenge [37]. A case series involving women with extra-tubal EP has also been published. Of the eight women included in the analysis, five had interstitial EPs, and the pregnancies of three women were located in the caesarean section scar. The combination of MTX and gefitinib proved to be effective in the above cases. Similar to previous reports, no severe side effects were noted [38].

Recently, a protocol of the multi-centre, double-blinded, placebo-controlled, randomized trial assessing the efficacy of combined therapy including gefitinib and methotrexate was developed. The results of this study may support the possible use of gefitinib for the treatment of EP. Currently, these results seem crucial in the daily clinical application of this method, since the number of patients included was significantly larger (328 patients) than in previous clinical trials [39].

Based on the analysed studies, it can be concluded that gefitinib in combination with MTX accelerates the resolution of an EP and reduces the risk of potential surgery. However, the overall assessment of this treatment should constitute some limitations of the above-mentioned studies. Firstly, all of them were conducted on a relatively small number of patients. Moreover, there are reports indicating that the use of gefitinib in the treatment of lung cancer has been associated with some severe adverse reactions including hepatotoxicity and interstitial lung disease. The risk of the occurrence of the latter, as the most serious, is strongly correlated with the coexistence of lung pathologies or Japanese ancestry [40,41]. Because of such a relation, the decision was made to exclude risk groups from these studies [35,36,39]. Thus, in the future, it is important to consider the occurrence risk of the severe side effects to constitute the exclusion criteria of patients.

The treatment methods from revised studies, including letrozole and gefitinib, are presented in Table 1.

## 6. Locally Administrated Substances

### 6.1. Absolute Ethanol

The effect of absolute ethanol (AE) injection in the treatment of EP is based on the rapid destruction of chorionic tissue surrounding the gestational sac in the mechanism of dehydration. An initial clinical trial with the use of AE in the therapy of EP was conducted on the group of 69 patients who experienced such a condition as a result of assisted reproductive techniques (ART). The rapid onset of the response to the treatment was listed as the greatest strength of this therapy. In this study, the efficacy of this method reached 87% [42]. A similar level of therapy efficiency of approximately 92% was observed in a study of 242 patients conducted by Osada et al. Moreover, they demonstrated the total effectiveness of AE injection in EPs with a foetal heartbeat [43].

A recent report from 2022 presents two patients who developed cervical EP through assisted reproductive technology. The patients were treated with the use of transvaginal ultrasound-guided local injections of absolute ethanol. In both cases, the treatment was successful and uneventful. The authors emphasize that local injections of AE may avoid the complications of MTX therapy or uterine artery embolization and may be an option for patients who desire fertility preservation [44].

It seems that this method may also have a satisfactory effect on the treatment of heterotopic pregnancies. In a study by Liu et al., the resolution of four out of five heterotopic pregnancies was achieved, but due to the small number of patients in the group, this treatment cannot be considered promising until further studies are available [45].

Lu et al., Kakinuma et al., and Osada et al., reported on the effectiveness of the use of AE injections in the treatment of caesarean section scar pregnancy (CSSP). Taking the difficulties of CSSP management into consideration, it is important to seek novel therapeutic options [46,47,48].

So far, only one study comparing the use of AE injection and surgical methods in the treatment of EP has been published. The authors observed that the use of a local injection of AE positively correlated with the future fertility of the patients, probably due to the lower invasiveness of the treatment. However, surgical management was characterized by a generally higher efficacy and shorter hospital stay [49].

Almost all studies discussing the use of AE injection presented consistent results, highlighting the essential benefits of this treatment model, including the low risk of infection due to the antiseptic properties of ethanol; rapid effect, which can be observed within two hours of administration; and low application costs [42,43,47,49]. The detailed results of analysing studies are presented in Table 2.

Nevertheless, an optimal clinical randomized study is yet to be conducted to confirm previous data.

### 6.2. Potassium Chloride (KCl)

KCl may be used to cease embryo or foetus cardiac activity, and consequently to cause foetal death [50,51]. In 1997, Godoin et al. successfully treated one patient with pregnancy in the caesarean section scar by the local use of KCl and MTX [50]. In the study conducted by Agarwal et al., 11 patients with EP in the caesarean section scar were described. Three of these cases presented foetal heart activity and received KCl and MTX locally as a treatment option. The remaining eight patients were classified to surgery (four hemodynamically unstable patients) or MTX treatment (four patients). In the KCl and MTX treated group, two patients developed uterine arteriovenous malformation and one was treated without complications [51]. Therefore, KCl with MTX may be effective in the case of a cervical pregnancy [52]. In a case report described by Petousis et al., there was one patient with cervical pregnancy treated by an intramuscular and intra-amniotic injection of MTX and KCl, respectively. The treatment was effective, and no other interventions were implemented [52]. The use of KCl may also be useful in the treatment of heterotopic pregnancy [53]. Habana et al. reviewed medical treatment with KCl and with or without MTX in heterotopic cornual pregnancies. All of them were successfully treated with no further need for surgical interventions. However, only 50% of intrauterine pregnancies resulted in live births [54]. All studies presenting the results of KCl treatment are presented in Table 3. 

## 7. Discussion

The question in focus in the present review concerned the issue of whether other new medications can change the gold standard of EP treatment—that is, whether they can replace the MTX treatment.

One of the newest drugs under examination includes aromatase inhibitors, especially letrozole. Mitwally et al. showed that letrozole treatment in tubal EP had the same efficiency as the MTX group. Moreover, β-hCG levels started to decline more rapidly and adverse effects were less common in the letrozole group than in the MTX group [22,27,28].

Moreover, other aromatase inhibitors could be implemented into research on ectopic pregnancy due to the same mechanism of action. Treatment with letrozole should also be compared with a multi-dose regime with methotrexate, as it may be slightly but significantly more successful than a single-dose one. Since ectopic pregnancies treated with letrozole were early and with low β-hCG levels, it should also be compared with expectant management [13].

Another promising substance in EP treatment is gefitinib, an inhibitor of EGFR tyrosine domain. Considering that the expression of EGFR in placental tissue is higher than in other non-malignant tissues, the use of gefitinib seems to be an interesting option [33,34]. Preclinical studies showed that gefitinib in a combination with MTX inhibited the growth of placental cells more effectively than when used separately [32]. In clinical trials, the effectiveness of such combined therapy was noticed to be between 85% and 86%, which provide a basis for the assumption that this treatment may change basic MTX treatment [35,36]. Despite the high efficacy, which outweighs the efficacy of MTX used separately, the combination therapy is not free of side effects, as observed in some studied patients [35,36,38]. As indicated by the authors, these side effects may be acceptable to patients, especially if treatment with gefitinib results in a shortening of therapy duration, but their assessment of the tolerability of these adverse reactions is unknown and further studies are needed.

So far, there are no studies, in which gefitinib is planned to be used alone, without MTX. Therefore, adverse effects connected to the MTX treatment still may be observed. Yet, it should not be forgotten that in many cases the usage of the second drug is helpful in reducing the dose of the first drug, and, consequently, the number of adverse effects and toxicity.

Even if the replacement of MTX into another drug is not possible, the reduction in the dose of orally administered drugs or the local administration of substances via injections may improve the outcome of treatment. Therefore, the analysis of the impact of locally administered substances such as KCl and absolute ethanol in EP was conducted. Most studies referred to AE as an effective treatment option, which is characterized by rapid metabolism, low toxicity, and low dosage [46]. Importantly, when compared to surgical treatment, this new method positively correlated with the future fertility of the patients. Therefore, it might be used as a first line treatment for women who want to preserve fertility. Moreover, this method can, in some cases, completely eliminate the use of MTX. KCl also showed its efficiency in caesarean section scar pregnancies, especially in combination with MTX [51]. This substance was also applied successfully in the case of cervical pregnancy, constituting an interesting therapeutic option for this type of EP [52].

An important limitation of the aforementioned studies is a small number of participants in the study groups. Some case reports were described which may become an indication for further studies performed on a greater number of patients. In addition, we cannot deny that the follow-up period after the administration of described substances was short; therefore, those substances should be examined and described in long-term observations.

## 8. Conclusions

As addressed in the present article, the treatment options for ectopic pregnancy should depend on its location. Different options should be selected to treat different ectopic pregnancies. EPs in a caesarean section scar or in the cervix are more accessible for the implementation of locally administered substances and avoid systemic adverse events. The tubal ectopic pregnancies are less available, but new medications including letrozole and gefitinib (even if used in connection with MTX) may reduce the number of adverse events, increase effectiveness, and change the actual gold standard. There is not enough evidence to prove that the presented methods of treatment are efficient, but previously conducted studies seem promising.

## Figures and Tables

**Table 1:** Review of the literature describing letrozole and gefitinib treatment in EP.

References	Number of Patients	Type of EP	Medical Treatment	Dose	Pre-Treatment β-hCG	Successfully Terminated	Time to Resolution
Mitwally et al., 2020 [22]	14	tubal EP	letrozole	5 mg daily (in 2 doses) for 10 days	mean 1065 mIU/mL (491.5–1438 mIU/mL)	12/14 (86%)	no data
Kochhar et al., 2021 [27]	1	tubal EP	letrozole	7.5 mg daily for 4 days 5 mg daily for 3 days	1525.51 mIU/mL	1/1 (100%)	no data (discharged after 8 days)
El-Sayed et al., 2022 [29]	25	tubal EP	letrozole	5 mg daily (in 2 doses) for 10 days	1134 mIU/mL	86%	no data
Skubisz et al., 2013 [35]	12	tubal EP	Gefitinib + MTX	250 mg daily (for 1, 3 or 7 days) + 50 mg/m^2^ i.m (one dose)	1000–3000 mIU/mL	10/12 (85%)	36.5 ± 12.54 days
Skubisz et al., 2018 [36]	28	tubal EP	Gefitinib + MTX	250 mg daily for 7 days + 50 mg/m^2^ i.m (one dose)	mean 2039 mIU/mL (1031–8575 mIU/mL)	24/28 (86%)	18–67 days (mean: 32 days)
Horne et al., 2014 [38]	8	5 interstitial EPs 3 caesarean scar EPs	Gefitinib + MTX	250 mg daily for 7 days + 50 mg/m^2^ i.m (one or two doses)	2458–48,550 IU/L	8/8 (100%)	25–196 days (mean: 65 days)

**Table 2:** Review of the literature describing absolute ethanol treatment in EP.

References	Number of Patients	Type of EP	Medical Treatment	Dose	Pre-Treatment β-hCG	Successfully Terminated	Time to Resolution
Kaijima et al., 2006 [42]	69	66 tubal EPs 1 interstitial EP 2 cervical EPs	AE	0.3 mL of AE	no data	(60/69) 87%	no data
Osada et al., 2020 [43]	242	200 tubal EPs 19 cervical EPs 17 interstitial EPs 3 caesarean scar EPs 3 peritoneal EPs	AE	average dose 3.2 mL in average 1.6 injections (range: 1–5)	mean 7034.6 mIU/mL (range: 347–135,040 mIU/mL)	222/242 (92%)	no data
Kakinuma et al., 2022 [44]	2	cervical EP	AE	5 mL (in two doses) and 4 ml	16,346 mIU/mL 26,930 mIU/mL	2/2 (100%)	17 and 10 days
Liu et al., 2019 [45]	5	4 tubal EPs 1 cervix EP (heterotopic)	AE	1.0–2.5 mL of AE 1–2 injections	range: 742.47–4066.00 mIU/mL	4/5 (80%)	no data
Lu et al., 2019 [46]	26	caesarean scar EP	AE	4.0 and 30 mL (mean 11.15 ± 6.37 mL) 1, 2 or 3 injections	mean 35,640 mIU/mL range: 10,801–93,544 mIU/mL	26/26 (100%)	36.5 ± 12.5 days
Kakinuma et al., 2021 [47]	1	caesarean scar EP	AE	1.2 mL of AE	91,798 mIU/mL	1/1 (100%)	no data (discharged by 7 days)
Osada et al., 2019 [48]	19	16 cervical EPs 3 caesarean scar EPs	AE	between 1.0 and 10.0 mL (average, 4.82 mL) 1–5 injections	mean 18,938 mIU/mL range: 3577–135,040 mIU/mL	19/19 (100%)	no data
Bi et al., 2019 [49]	55	tubal EP	AE	no data	1641.92 ± 2068.00 mIU/L	46/55 (84%)	no data

**Table 3:** Review of the literature describing KCl treatment in EP.

Reference	Patients	Type of EP	Medical Treatment	Dose	Pre-Treatment β-hCG	Successfully Terminated	Time to Resolution
Agarwal et al., 2021 [51]	3	caesarean scar EP	KCl + MTX	1–2 mEq KCl, no more data	7614, 38,444, 56,860 mIU/mL	3/3 (100%)	61, 92, 106 days
Petousis et al., 2015 [52]	1	cervical EP	KCl + MTX	2 mEq/mL KCl + 50 mg/m^2^ i.m MTX	28,590 IU/L	1/1 (100%)	56 days
Habana et al., 2000 [54] (review)	10	heterotopic EP	KCl + MTX	1–2 mL 2 mEq KCl	no data	10/10 (100%)	No data

## Data Availability

Not applicable.

## Supplementary Materials

The following supporting information can be downloaded at: https://www.mdpi.com/article/10.3390/ijerph192114230/s1.
