# Supplementary material for: Future Perspectives of Ectopic Pregnancy Treatment—Review of Possible Pharmacological Methods
Source: Int J Environ Res Public Health. 2022 Oct 31;19(21):14230. doi: 10.3390/ijerph192114230 (PMC9656791; doi:10.3390/ijerph192114230)
Supplement: Supplementary file 1 [file ijerph-19-14230-s001.zip › ijerph-1965025-supplementary.pdf]

Supplenetary materials:

Materials and methods

A literature search was carried out in the PubMed, Google Scholar and Scopus databases between November 2021 and October 2022. We used the combination of terms concerning ectopic pregnancy including "ectopic pregnancy","tubal ectopic pregnancy", "interstitial ectopic pregnancy" or "caesarean scar ectopic pregnancy" with the following search terms: "treatment", "medical treatment", "new treatment options", "methotrexate", "MTX", "aromatase inhibitors", "letrozole", "gefitinib", "absolute ethanol", "KCl" and "potassium chloride". All combinations of terms used in the article selection process were listed in the following table.

FIRST COMPONENT OF THE SEARCHED TERMS	SECOND COMPONENT OF THE SEARCHED TERMS
ectopic pregnancy	treatment
tubal ectopic pregnancy	medical treatment
interstitial ectopic pregnancy	new treatment options
caesarean scar ectopic pregnancy	methotrexate
	MTX
	aromatase inhibitors
	letrozole
	gefitinib
	absolute ethanol
	KCl
	potassium chloride

Inclusion criteria:

- no limitations of the year of published study were applied
- types of studies?
- study cases

Exclusion criteria:

- articles not written in English
- conference abstracts only
- duplicated papers

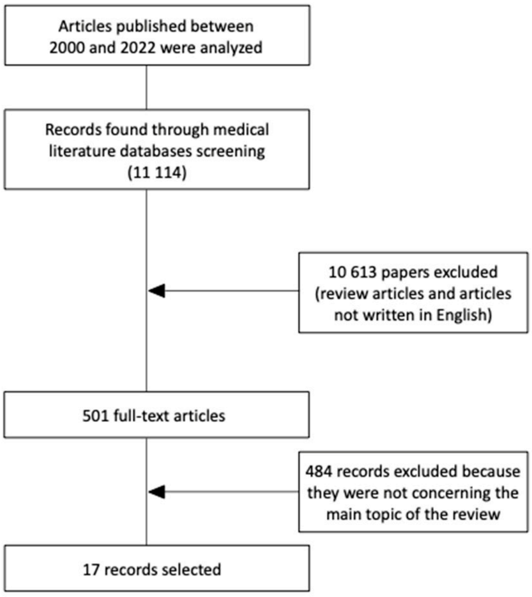
Articles published between 2000 and 2022 were analyzed
Records found through medical literature databases screening (11 114)
10 613 papers excluded (review articles and articles not written in English)
501 full-text articles
484 records excluded because they were not concerning the main topic of the review
17 records selected
